# Inositol Alleviates Pulmonary Fibrosis by Promoting Autophagy via Inhibiting the HIF-1*α*-SLUG Axis in Acute Respiratory Distress Syndrome

**DOI:** 10.1155/2022/1030238

**Published:** 2022-12-23

**Authors:** Yufeng Liang, Yingyi Xu, Bingtai Lu, Yuanming Huang, Shuman Xu, Junjie Xie, Ming Liu, Di Che, Liuheyi Ma, Jianping Tao, Jie Hong, Jianhui Zhang, Xun Situ, XinXu Ou, Lihe Chen, Yang Li, Lihong Zhang, Zhiyuan Wu

**Affiliations:** ^1^Pediatric Intensive Care Unit, Guangzhou Women and Children's Medical Center, Guangzhou Medical University, Guangzhou, Guangdong, China; ^2^Department of Pediatrics, Linzhi People's Hospital, Linzhi, Tibet, China; ^3^Department of Anaesthesiology, Guangzhou Women and Children's Medical Center, Guangzhou Medical University, Guangzhou, Guangdong, China; ^4^Guangdong Provincial People's Hospital, Guangdong Academy of Medical Sciences, Guangzhou, China; ^5^Guangzhou Institute of Pediatrics, Guangzhou. Women and Children's Medical Center, Guangzhou Medical University, Guangzhou, Guangdong, China; ^6^Medical Management Department of Guangdong Province Hospital for Women and Children Healthcare, Guangzhou, Guangdong, China; ^7^Maternal and Child Health Hospital of Sanshui District, Foshan, Guangdong, China; ^8^Library, Guangzhou Women and Children's Medical Center, Guangzhou Medical University, Guangzhou, Guangdong, China

## Abstract

The effective remission of acute respiratory distress syndrome- (ARDS-) caused pulmonary fibrosis determines the recovery of lung function. Inositol can relieve lung injuries induced by ARDS. However, the mechanism of myo-inositol in the development of ARDS is unclear, which limits its use in the clinic. We explored the role and mechanism of myo-inositol in the development of ARDS by using an *in vitro* lipopolysaccharide- (LPS-) established alveolar epithelial cell inflammation model and an *in vivo* ARDS mouse model. Our results showed that inositol can alleviate the progression of pulmonary fibrosis. More significantly, we found that inositol can induce autophagy to inhibit the progression pulmonary fibrosis caused by ARDS. In order to explore the core regulators of ARDS affected by inositol, mRNA-seq sequencing was performed. Those results showed that transcription factor HIF-1*α* can regulate the expression of SLUG, which in turn can regulate the key gene *E-Cadherin* involved in cell epithelial-mesenchymal transition (EMT) as well as N-cadherin expression, and both were regulated by inositol. Our results suggest that inositol activates autophagy to inhibit EMT progression induced by the HIF-1*α*/SLUG signaling pathway in ARDS, and thereby alleviates pulmonary fibrosis.

## 1. Introduction

Acute respiratory distress syndrome (ARDS) is a type of acute diffuse, inflammatory lung injury, leading to increased pulmonary vascular permeability, increased lung weight, and loss of aerated lung tissue. The morphological hallmark of the acute phase is diffuse alveolar damage (i.e., edema, inflammation, hyaline membrane, or hemorrhage) [1, 2]. ARDS has become one of the most common serious diseases seen in the clinic; it has a rapid onset and high rates of morbidity and mortality [3]. While great progress has been made in the treatment of ARDS with anti-inflammatory drugs and assisted mechanical ventilation, the mortality rate of patients with severe ARDS remains at 70% [4]. Therefore, it is important to find new therapeutic drugs or methods that can improve the treatment and prognosis of patients with ARDS.

Inositol (also known as Myo-inositol, hexahydroxycyclohexane, or cis-1,2,3,5-trans-4,6-cyclohexanehexol) is a six carbon cyclitol that contains five equatorial hydroxyl groups and one in axial position. The main source of myo-inositol is the diet; indeed, it is found in a wide variety of foods such as whole grains, seeds, and fruits. Inositol can also be synthesized from glucose, the immediate precursor being fructose 6-phosphate, which is converted to myo-inositol by a cyclase. Inositol is a precursor in the phosphatidylinositol cycle [5]. Inositol promotes maturation of the surfactant phospholipids phosphatidylcholine and phosphatidylinositol, and it or its derivatives can serve as important intracellular signaling molecules that regulate organ function [6]. Myo-inositol increases the synthesis and function of the surfactant phosphatidylinositol by acting on the phosphoinositide 3-kinase- (PI3K-) regulated signaling pathway and reducing immune function and oxygenation at the bronchoalveolar level [7]. It was recently shown that myo-inositol can decrease IL-6 expression by regulating the IRE1 and STAT3 pathways, and thereby improve oxygenation in patients with severe ARDS caused by SARS-CoV-2 infection [8]. In addition, it was proven that inositol derivatives and phosphatidylglycerol subfractions in surfactant preparations can mitigate key inflammatory pathways in a neonatal piglet ARDS model, suggesting potential therapeutic roles for surfactant-inositol [9]. However, the mechanism by which inositol affects ARDS remains unclear.

The pathogenesis and pathophysiological processes of ARDS are very complicated and have not been fully elucidated. However, most investigators believe that an “out of control” inflammatory response, excessive rates of cell damage and repair, and abnormal cell apoptosis and proliferation all contribute to the development of ARDS [10]. Autophagy is a process involving the self-degradation of cellular organelles and proteins and plays important roles in cell differentiation, survival, and homeostasis [11]. Increasing numbers of studies have shown that autophagy also plays an important role in many pathophysiological conditions, such as cancers, infections, heart disease, and acute lung injuries (ALIs). For example, autophagy was found to reduce inflammasome activity and IL-1 beta levels in LPS plus mechanical ventilation-induced ALIs by preventing the development of increased lung permeability and hypoxemia [12]. It was also shown that penehyclidine hydrochloride can alleviate LPS-induced ARDS in cells by regulating autophagy-related pathways [13]. Epithelial-mesenchymal transition (EMT) is the transition of epithelial cells to mesenchymal cells under either normal physiological or special conditions [14]. The EMT process plays an important role in regulating malignant biological behavior by promoting tumor cell invasion and migration [15]. It also plays an important role in ventilation-associated lung fibrosis, which may contribute to the poor outcomes of patients with acute respiratory distress syndrome [16]. When taken together, the above findings suggest that both autophagy and EMT play crucial roles in the development of ARDS.

It has been demonstrated that autophagy plays important roles in different diseases via their synergism or antagonism and especially in cancers. It was recently demonstrated that autophagy inhibition-mediated EMT augments local myofibroblast differentiation in pulmonary fibrosis, suggesting that autophagy and EMT might both participate in regulating ARDS progression. However, it remains unclear whether the role played by inositol in ARDS is related to autophagy. Therefore, we explored the mechanism by which inositol regulates ARDS and sought to determine whether inositol could alter autophagy both *in vitro* and *in vivo*.

## 2. Materials and Methods

### 2.1. Cell Culture and Treatment

Human pulmonary alveolar epithelial cells (HPAEpiCs) (BNCC337859) were purchased from the BeNa culture collection (Beijing, China) and cultured in Dulbecco's modified eagle medium (DMEM) supplemented with 10% fetal bovine serum (FBS) in an atmosphere consisting of 95% air and 5% CO_2_ at 37°C. HPAEpiCs were treated with lipopolysaccharide (LPS) for 24 h to establish an ARDS model. For inositol treatment, LPS-incubated HPAEpiCs were treated with 10 *μ*g/mL inositol for 24 h. For rapamycin (RAPA) and 3-Methyladenine (3-MA) treatment, LPS-incubated HPAEpiCs were incubated with 10 nM rapamycin (AY-22989, Sirolimus) plus 5 mM 3-MA (S2767, Selleck, Houston, TX, USA) for 2 h and then collected for further analysis.

### 2.2. Enzyme-Linked Immunosorbent Assay (ELISA)

Samples of mouse blood serum and the supernatants of HPAEpiCs were collected for analysis of cytokines, including Interleukin 1 Beta (IL-1*β*), Interleukin 6 (IL-6), Interleukin 17 (IL-17), and monocyte chemotactic protein 1 (MCP-1) by using Mouse IL-1*β* (E-EL-M0037c, Elabscience, China), IL-6 (E-EL-M0044c, Elabscience), IL-17 (3020092, BioAim Scientific Inc., Toronto, Canada), and MCP-1 (E-EL-M3001, Elabscience) ELISA kits according to the manufacturer's instructions.

### 2.3. mRNA-Seq Analysis

Total RNA was extracted using TRIzol Reagent (15596-018, Life Technologies, Carlsbad, CA, USA) according to the manufacturer's instructions. RNA integrity was evaluated with an Agilent Bioanalyzer 2100 (Agilent Technologies, Santa Clara, CA, USA), and the RNA was then purified using a Clean XP Kit (A63987, Beckman Coulter, Indianapolis, IN, USA). An mRNA library was established using a VAHTS Universal V6 RNA-seq Library Prep Kit for Illumina® (NR604-02, Vazyme), and mRNA sequencing was performed on an Illumina Hiseq 2000 platform. The differentially expressed genes (DEGs) were analyzed using edgeR software. The cutoff values for differential expression were a *q* value ≤ 0.05 and fold − change ≥ 2. The DEGs are shown on a Heatmap and Volcano map.

### 2.4. Reverse Transcription Quantitative PCR (RT-qPCR)

The total RNA was extracted from HPAEpiCs by using TRIzol reagent according to the manufacturer's instructions (15596026, Thermo Fisher, Waltham, MA, USA), and then reverse transcribed to cDNA using 5x primeScript RT Master Mixperfect for Real Time PCR (Takara, Tokyo, Japan). qRT-qPCR was performed by using Bestar® SybrGreen qPCR Master Mix (DBI) on an Agilent Stratagene Mx3000P PCR system. The conditions used for qRT-qPCR were as follows: 95°C for 2 min; 40 cycles of 95°C for 20 s, 58°C for 20 s, and 72°C for 20 s. The 2^−△△ct^ method was used to calculate the relative expression levels of the target gene. GAPDH served as a reference.

### 2.5. Western Blot Analysis

The total proteins were extracted from HPAEpiCs or lung tissues by using lysis buffer (P0013G, Beyotime, China), and the protein concentration in each extract was determined using a Bradford protein assay kit (ab102535, Abcam, Cambridge, UK. Next, a 20 *u*g sample of total protein from each extract was separated by SDS-PAGE (10%; Beyotime), and the protein bands were transferred onto PVDF membranes (Millipore, Billerica, MA, USA), which were subsequently incubated overnight at 4°C with the following antibodies: anti-LC3B (1 : 1000, ab51520, Abcam, Cambridge, MA, USA), P62 (1 : 1000, ab91526, Abcam), Beclin-1 (1 : 1000, ab207612, Abcam), SLUG (1 : 1000, ab51772, Abcam), HIF-1*α* (1 : 500, ab92498, Abcam), E-cadherin (1 : 500, ab40772, Abcam), N-cadherin (1 : 1000, ab202030, Abcam), and GAPDH (1 : 10000, ab8245, Abcam). Next, the membranes were incubated with an HRP goat anti-rabbit/mouse IgG secondary antibody (1 : 20000, ab8245, Abcam); after which, the immunostained protein bands were detected using an enhanced chemiluminescence detection system (Millipore). The integral optical density values of the protein bands were analyzed using Image Pro Plus 6.0 software.

### 2.6. Animals and the ARDS Model

Mouse ARDS model as described previously [2]. C57BL/6 mice (*n* = 15; 6-8 weeks old; weight range 18-22 g) were purchased from the Experimental Animal Center of Southern Medical University and housed in an SPF animal laboratory with a humidity of 60%-65%, temperature range of 21-23°C and a 12 h light and dark cycle. The mice were randomly assigned to the following 3 groups (*n* = 5 per group): blank control group, model group, and a model with inositol group. The mice were anesthetized by an intraperitoneal injection of 3% pentobarbital sodium (50 mg/kg), and then given LPS (40 *μ*L, 5 mg/kg) via intranasal instillation. Inositol (60 mg/kg) was perfused into the trachea after 4 hours of infusion. After 72 hours, the animals were sacrificed with an overdose of pentobarbital (130 mg/kg) and lung injuries were detected by histopathological staining and measurements of cytokine levels. The protocols for all animal studies were approved by the Animal Ethics Committee of Guangzhou Yongnuo Medical Laboratory Animal Center.

### 2.7. Histopathological Staining

Specimens of lung tissue from mice in the different groups were fixed with 4% paraformaldehyde, embedded in paraffin, and then sliced into 4 *μ*m sections for hematoxylin-eosin (H&E) staining and Masson's staining. Briefly, the tissue slices were dewaxed in xylene, rehydrated by a graded alcohol series, and then stained with hematoxylin for 3-5 min. Next, the tissue slices were further stained with eosin for 4 min, washed with PBS, and cleared in a solution of 1% HCl in 70% alcohol. For Masson's staining, the tissue slices were fixed with neutral formalin, sectioned, deparaffinized in water, stained with Masson's composite staining solution for 5 min, and then washed with 0.2% acetic acid solution and 5% phosphotungstic acid for 5-10 min. The sections were then stained with bright green staining solution for 5 min, washed twice with 0.2% acetic acid solution, dehydrated in absolute alcohol, immersed in xylene for transparency, and mounted onto slides with neutral gum. For PAS staining, the tissue slices were fixed with neutral formalin, sectioned, deparaffinized in water, and then oxidized by treatment with 0.5% periodic acid aqueous solution for 10 min. Subsequently, the slices were stained with Schiff's reagent for 15-30 min, and hematoxylin was used to counterstain the nucleus for 1-2 min. The slices were then dehydrated in absolute alcohol, immersed in xylene for transparency, and mounted onto slides with neutral gum. Finally, the mounted tissue sections were observed under a light microscope.

### 2.8. Immunofluorescence Staining

The LC3B expression that contributes to autophagy was detected by immunofluorescence (IF). Briefly, 1 × 10^6^ HPAEpiCs were fixed with 4% paraformaldehyde for 30 min at room temperature in 24-well plates. Next, the cells were blocked with 10% goat serum for 15 min at 4°C and, subsequently, incubated with an LC3B antibody (1 : 200, ab192890, Abcam), SLUG antibody (1 : 300, ab224731, Abcam), and HIF-1*α* antibody (1 : 500, ab179483, Abcam). The cells were then incubated with the TRITC-conjugated secondary antibody Anti-Rabbit IgG H&L, (Alexa Fluor ® 647) (1 : 1000, ab15007, Abcam) and Goat Anti-Mouse IgG H&L (Alexa Fluor® 488, 1 : 1000, ab150113, Abcam) for 1 h at 37°C in the dark. After being counterstained with 4′,6-diamidino-2- phenylindole (DAPI, 0.1 *μ*g/mL; Sigma Aldrich, St. Louis, MO, USA) for 5 min, the cells were photographed using a fluorescent microscope at 647 nm (OLYMPUS, IX71).

### 2.9. Transmission Electron Microscopy (TEM)

Autophagy was detected by TEM as described in a previous report [17]. Briefly, HPAEpiCs were fixed in 2.5% glutaraldehyde solution for 2 h and then rinsed with precooled 0.1 M PBS buffer (pH 7.0). Next, the cells were washed with buffer and then fixed with 1% osmium tetroxide and 0.1 M cacodylate buffer (pH 7.4). Gradient dehydration was performed by using different concentrations of ethanol after washing with the buffer solution. The cells were then embedded in epoxy resin, and the ultrastructure of cells undergoing autophagy was observed and photographed under a transmission electron microscope (JEM-1200, Japan) operated at 80 kV.

### 2.10. Luciferase Reporter Assay

The interaction between HIF-1*α* and its *SLUG* target gene was predicted using the JASPAR database (http://jaspar.binf.ku.dk/) and, subsequently, validated using the dual-luciferase reporter assay. Briefly, the promoter sequence of SLUG carrying the wild type and mutant binding sites was cloned into the dual-luciferase reporter vector, pGL3-basic. The full length sequence of HIF-1*α* was cloned into the pcDNA3.0 vector to force overexpression of HIF-1*α*. The SLUG-pGL3-basic and HIF-1*α* sequences was cotransfected into cells using Lipofectamine 2000. Relative luciferase intensities were detected with a microplate reader at 48 h posttransfection according to instructions provided by the manufacturer of the luciferase reporting system (Promega, Mannheim, Germany).

### 2.11. Chromatin Immunoprecipitation (ChIP) PCR Analysis

ChIP-PCR was performed as described in a previous report [18]. Briefly, cultured cells at 60-80% confluence were crosslinked by incubation with 1% formaldehyde at 37°C for 10 min. Next, the cells were treated with 400 *μ*L of SDS lysis buffer, followed by ultrasonic disruption (VCX750) for 20 min (14 cycles of 4.5 s ultrasound administered at 9 s intervals). The disrupted cells were centrifuged at 10,000 g for 10 min; after which, 0.2 M NaCl was added to the mixture, which was then incubated for 3 h at 65°C to decrosslink. The supernatant was diluted with 900 *μ*L of ChIP dilution buffer and 20 *μ*L of 50 × PIC and then incubated overnight at 4°C with 100 *μ*L of anti-HIF-1*α* antibody and protein G beads. Samples were collected and washed two times in lysis buffer and four times in 1 M lysis buffer; after which, the beads were resuspended in lysis buffer and treated with proteinase K for 45 min 45°C. Coprecipitated DNAs were purified using a QIAquick DNA purification spin column (Qiagen, Germantown, MD, USA) and eluted with 50 *μ*L of nuclease-free water. The immunoprecipitated DNA was quantified by PCR performed in a 20 *μ*L volume. The primers used for PCR were as follows: SLUG-F: 5′-TGTCCGGTGGTTCCAAATGA-3′; SLUG-R: 5′-CTGCGCTACTCAGGGCTTC-3′.

### 2.12. Statistical Analysis

All statistical analyses were performed using IBM SPSS Statistics for Windows, Version 23.0 (IBM Corp., Armonk, NY, USA), and results are presented as the mean value ± SD of data obtained from least three independent experiments. Differences between two groups and more than three groups were analyzed using the unpaired *t*-test or one-way ANOVA followed by Tukey's multiple comparisons test, respectively. A *p* value < 0.05 was considered to be statistically significant.

## 3. Results

### 3.1. Inositol Attenuated Lung Injuries in the ARDS Model Mice by Activating Autophagy

**Figure 1:**
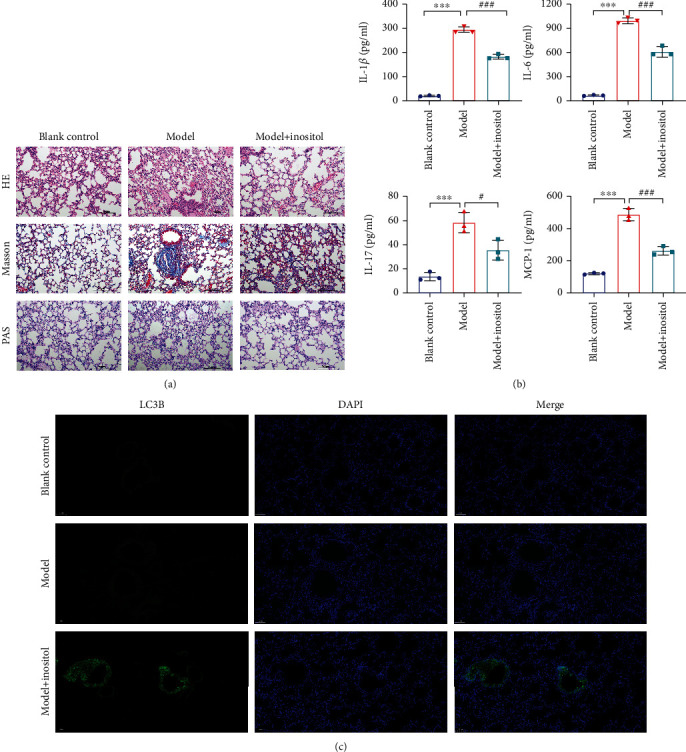
Inositol attenuated lung injuries in ARDS model mice by activating autophagy. (a) H&E and Masson's staining analysis of lung tissues from the ARDS model mice and the effects of inositol treatment, magnification, ×200. (b) The levels of IL-1*β*, IL-6, IL-17, and MCP-1 in the serum of mice from different groups were detected by ELISA. (c), LC3B immunofluorescence staining was performed to detect autophagy changes in ARDS model mice and the effects of treatment with inositol. Data represent the mean value ± SD, ^∗^, Model vs. Blank: ^∗∗∗^*p* < 0.001; ^#^, Model with Inositol vs. Model: ^#^*p* < 0.05; ^###^*p* < 0.001.

An ARDS model was established and used to evaluate the effect of inositol on lung injuries. H&E staining showed that when compared with mice in the blank control group (BC), the lung tissues of mice in the model group showed signs of massive inflammatory cell infiltration, pulmonary hemorrhage, and pulmonary interstitial fibrosis (Figure 1(a)). Masson's staining showed that collagen deposition occurred not only in the basement membranes of bronchial epithelia and blood vessels but also in lung parenchyma, indicating increased fibrosis in the lung. PAS staining showed that the numbers of PAS+, mucus-containing, metaplastic goblet cells were significantly increased in the model group. However, the degrees of inflammatory cell infiltration, pulmonary hemorrhage, collagen deposition, and the numbers of PAS+, mucus-containing, metaplastic goblet cells in the ARDS model mice could be greatly reduced by treatment with inositol (Figure 1(a)**)**. In order to further confirm the effect of inositol on ARDS, the levels of cytokines in the serum of mice in the different groups were examined by ELISA. As shown in Figure 1(b), the levels of IL-1*β*, IL-6, IL-17, and MCP-1 were significantly higher in the model group than in the blank control group. Surprisingly, those increases in the model group were attenuated by treatment with inositol. Simultaneously, LC3B IF staining showed that the fluorescence signal was stronger in the inositol treatment group than in the model group (Figure 1(c)**)**.

### 3.2. Inositol Attenuated Cytokine Release in the ARDS Cell Model by Activating Autophagy

**Figure 2:**
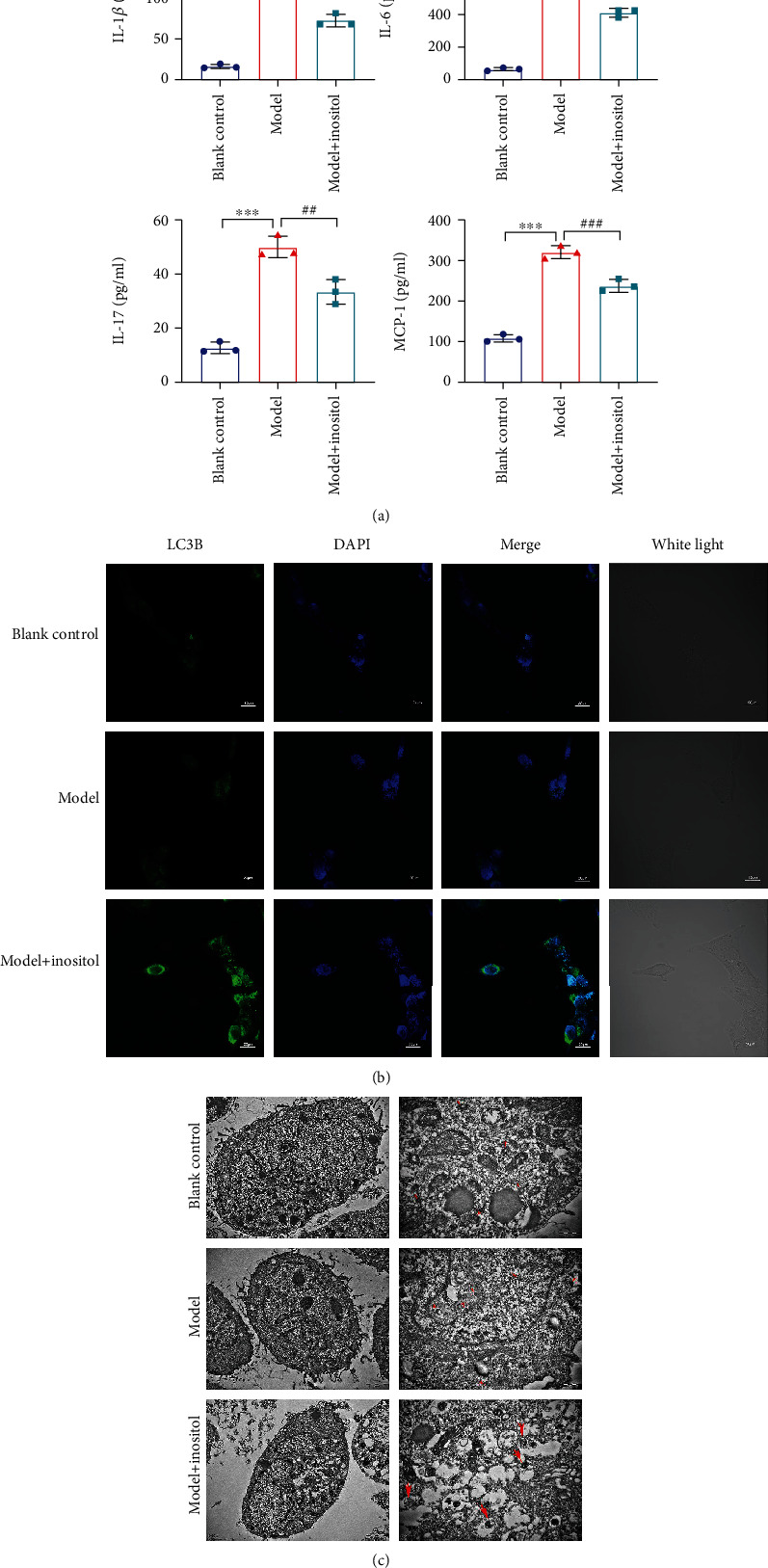
Inositol attenuated the release of cytokines from ARDS model cells by activating autophagy. (a) The levels of IL-1*β*, IL-6, IL-17, and MCP-1 in LPS-induced HPAEpiCs with and without inositol treatment were detected by ELISA. (b) LC3B immunofluorescence staining was performed to detect autophagy changes in LPS-induced HPAEpiCs with and without inositol treatment, Bar = 20 *μ*m. (c) Autophagosome changes in HPAEpiCs from the different groups as detected by TEM, Bar = 2 *μ*m. Data represent the mean value ± SD, ^∗^, Model vs. Blank: ^∗∗∗^*p* < 0.001; ^#^, Model with Inositol vs. Model: ^##^*p* < 0.01, ^###^*p* < 0.001.

In order to further confirm that inositol attenuated lung injuries in the ARDS model mice by activating autophagy, HPAEpiCs were incubated with LPS to establish a cell model. As shown in Figure 2(a), the levels of IL-1*β*, IL-6, IL-17, and MCP-1 in the LPS-induced HPAEpiCs (model group) were obviously higher than those in the blank cells. However, the increases in those cytokine levels could be attenuated by treatment with inositol. In addition, LC3B immunofluorescent staining showed that when compared with the blank control group, the fluorescence signal in HPAEpiCs induced with LPS showed no significant change. However, after the LPS-incubated HPAEpiCs (model group) were treated with inositol, the LC3B fluorescence signal was obviously enhanced, **(**Figure 2(b) and Supplementary Figure 1A), suggesting that inositol could activate autophagy in LPS-induced HPAEpiCs to alleviate ARDS-caused lung injury. The autophagosome changes in HPAEpiCs from the different groups were further confirmed by bio-TEM. As shown in Figure 2(c), TEM observations showed that the numbers of autophagosomes in the LPS-induced HPAEpiCs (model group) were increased following treatment with inositol.

### 3.3. *SLUG* Was Identified as a Gene Affected by Inositol and Might Be Involved in the Development of ARDS

**Figure 3:**
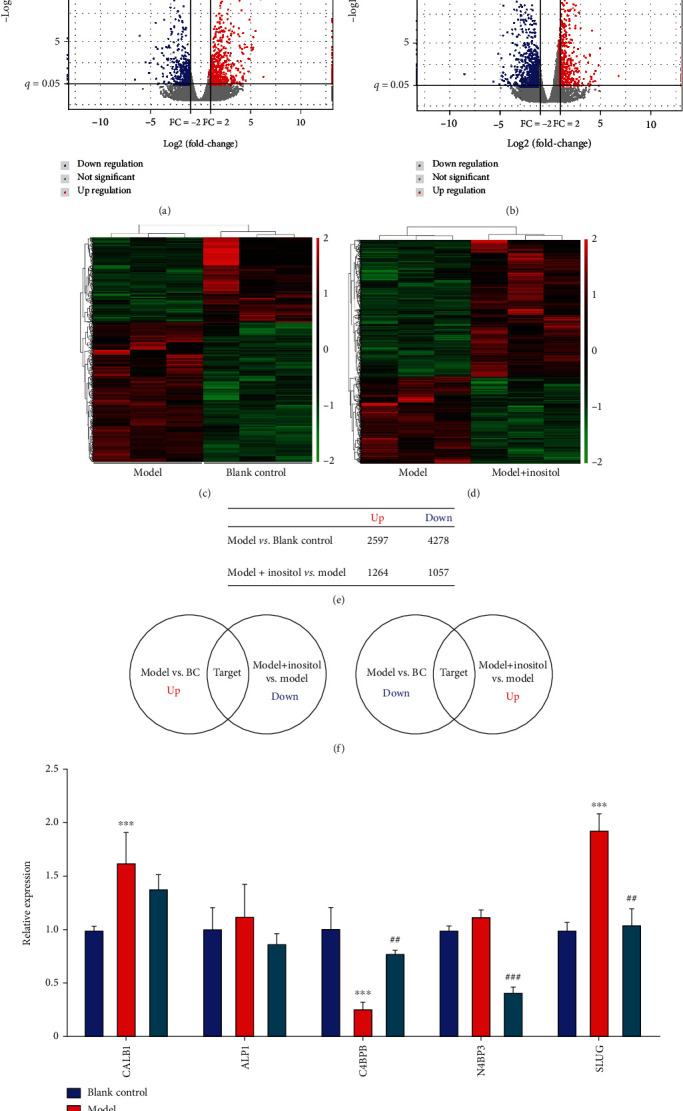
SLUG was affected by inositol and contributed to the development of ARDS. (a, b) Volcano map of genes that were differentially expressed in the model group vs. the blank control group. (c, d) Heatmap of genes that were differentially expressed in the model group vs. the blank control group and in the model + inositol group vs. the model group; *X* axis indicates the samples used for sequencing, and the *Y* axis indicates clusters of genes with similar expression patterns; green and red indicate the expression level from low to high. (e) The number of different genes expressed in the model group vs. the normal group, the model group vs. the inositol group, and the model + inositol group vs. the model group. (f) The Venn diagram shows the search for common genes that were differentially expressed in the model group vs. the normal group, the model group vs. the inositol group, and the model + inositol group vs. the model group. (g) The levels of *SLUG*, *C4BPB*, *CALB1*, N4BP3, and ALP1 expression were detected by qPCR; GAPDH served as a control. Data represent the mean value ± SD, ^∗^, Model vs. Blank: ^∗∗∗^*p* < 0.001; ^#^, Model with Inositol vs. Model: ^##^*p* < 0.01, ^###^*p* < 0.001.

In order to confirm the key factors affected by inositol that contribute to the development of ARDS, mRNA-seq was performed on HPAEpiCs cells after treatment with LPS alone or LPS plus inositol. The sequencing reads from different samples covered all 24 chromosomes, which mainly carried genes and coding regions. In addition, the number of genes became saturated as the sequencing volume increased (Figures 3(a) and 3(b), Supplementary Figure 2). As shown in Figures 3(c) and 3(d), when compared with the blank control group, 6875 differentially expressed genes (DEGs) were identified; among which, 2597 DEGs were upregulated, and 4278 DEGs were downregulated after treatment with LPS. When compared with the model group, a total of 2321 DEGs were found in the model group treated with inositol. Among those 2321 DEGS, 1264 were upregulated, and 1057 were downregulated (Figure 3(e)). A GO enrichment analysis showed that the genes with differential expression between the model and blank control group were mainly enriched in the isoprenoid biosynthetic process, cartilage morphogenesis, and retinoic acid metabolic process, while the genes with differential expression between the model with inositol group and model group were mainly enriched in sterol biosynthetic process, regulation of steroid biosynthetic process, and cholesterol biosynthetic process. A KEGG pathway analysis showed that the genes with differential expression between the model and blank control group were mainly involved in axon guidance, protein digestion and absorption, and inflammatory mediator regulation of TRP channels, while the genes with differential expression between the model plus inositol group and model group were mainly involved in the TNF signaling pathway, pertussis, and AGE-RAGE signaling pathway in diabetic complications (Supplementary Figure 3). To further explore the genes affected by inositol, we analyzed the number of genes that were altered (increased or decreased expression) due to the effects of LPS and inositol and screened for the number of genes that were coaltered (Figure 3(f)**)**. A total of 5 genes were identified which showed upregulation in the model group when compared with the blank control group but downregulation in the model with inositol group when compared with the model group; those 5 selected genes included *SLUG*, *C4BPB*, *CALB1*, *N4BP3*, and *ALP1*. The degrees of upregulation and downregulation of those 5 genes were further confirmed by qPCR. *C4BPB* was found to be downregulated in the model group, but its expression was restored to that of the control in the inositol group (Figure 3(g)). Among all the DEGS, *SLUG* showed the greatest fold changes in different groups and was, therefore, selected for further analysis.

### 3.4. Inositol Inhibited EMT by Downregulating SLUG Expression

**Figure 4:**
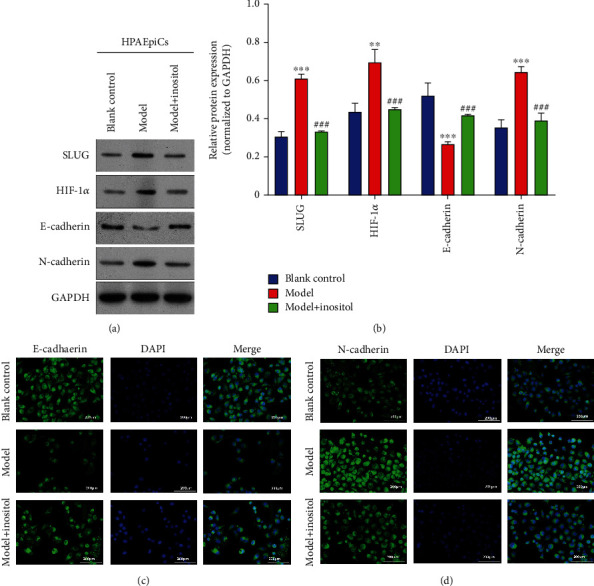
Inositol inhibited EMT by downregulating *SLUG*. (a) Western blot analysis for the expression of SLUG- and EMT-related proteins, including E-cadherin and N-cadherin; GAPDH served as a control. (b) Grey density was calculated using ImageJ software. (c, d) E-cadherin and N-cadherin expressions were detected by immunofluorescence staining; DAPI was used to stain the nucleus. Bar = 200 *μ*m. Data represent the mean value ± SD. ^∗^, Model vs. Blank: ^∗∗^*p* < 0.01, ^∗∗∗^*p* < 0.001; ^#^, Model with Inositol vs. Model: ^###^*p* < 0.001.

It has been demonstrated that SLUG plays key roles in the cellular EMT process that occurs in various diseases. In order to understand whether inositol inhibits EMT by downregulating SLUG, the expression of key proteins in the EMT process (E-cadherin and N-cadherin) was measured by western blotting and IF. Our western blot analyses showed that the levels of HIF-1*α*, SLUG, and N-cadherin protein expression were significantly elevated in the model group when compared with the blank control group. However, the increases in SLUG and N-cadherin expression induced by LPS were attenuated by treatment with inositol (Figure 4(a)). The changes in E-cadherin expression that occurred in the different groups were the opposite of those shown by SLUG and N-cadherin (Figure 4(a)). The IF analyses of E-cadherin and N-cadherin in the different groups further confirmed the changes in E-cadherin and N-cadherin expression (Figure 4(b) and Supplementary Figure 1B). At the same time, we performed IF experiments to detect the expression of E- and N-Cadherin. The results showed that inositol could increase E-Cadherin, reduce the expression of N-Cadherin, and play a role in inhibiting fibrosis **(**Figure 4(c)**)**.

### 3.5. *SLUG* Was a Direct Target of HIF-1*α*

**Figure 5:**
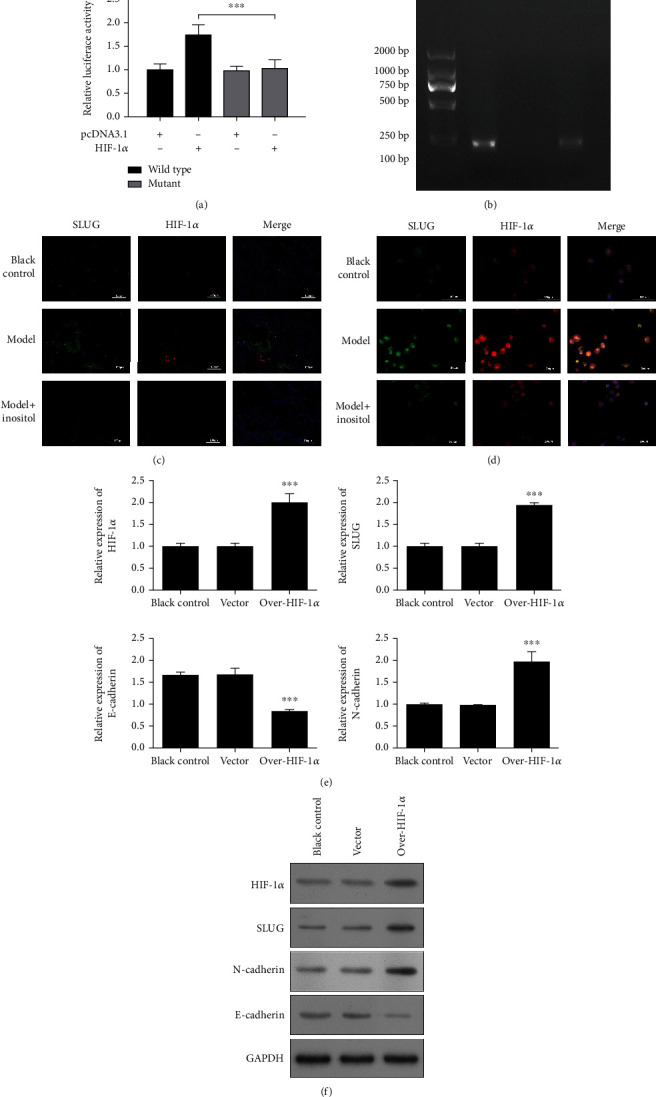
*SLUG* was a direct target of HIF-1*α*. (a) An HIF-1*α* binding site at loci 776-783 in the promoter region of *SLUG* was predicted by JASPAR. A dual luciferase reporter assay was performed to detect the regulation of *SLUG* by HIF-1*α*. Data represent the mean value ± SD, ^∗^, pcDNA-SLUG-WT+HIF-1*α* group vs. pcDNA-SLUG-WT group: ^∗∗∗^*p* < 0.001. (b) The binding function of HIF-1*α* to *SLUG* was analyzed by ChIP-PCR analysis; inositol served as a positive control, and IgG served as a negative control. (c, d) Colocalization of SLUG and HIF-1*α* in lung tissues and cells as detected by IF staining. (e) SLUG, HIF-1*α*, N-cadherin, and E-cadherin expression in cells with HIF-1*α* overexpression was detected by qPCR. (f) HIF-1*α*, SLUG, E-cadherin, and N-cadherin expression was detected by western blotting. Data represent the mean value ± SD, ^∗^, over-HIF-1*α* vs. vector: ^∗∗∗^*p* < 0.001.

The changes in HIF-1*α* and SLUG expression appeared to be highly correlated, and we, therefore, speculated that there might be a regulatory relationship between HIF-1*α* and SLUG. A further analysis conducted using JASPAR (http://jaspar.genereg.net/) revealed an HIF-1*α* binding site at loci 776-783 in the promoter region of *SLUG* (Figure 5(a)), suggesting that HIF-1*α* might regulate *SLUG* by binding to its promoter region. A dual luciferase reporter assay showed that the relative luciferase activity in the *SLUG* wild type (WT) group was significantly elevated after cotransfection with HIF-1*α* when compared with luciferase activity in the blank control group, while no changes were detected in the SLUG mutant group (Figure 5(a)), indicating that HIF-1*α* could bind to the SLUG promoter region. A ChIP-PCR analysis showed that when compared with the IgG group, the target band was amplified in the anti-HIF-1*α* group (Figure 5(b)), suggesting that the target sequence containing binding sites was successfully enriched by anti-HIF-1*α*. Furthermore, an IF staining analysis of SLUG and HIF-1*α* proteins in lung tissues showed that the red and green fluorescence signals which indicate HIF-1*α* and SLUG, respectively, were overlapped (Figure 5(c)). A further IF analysis of the cells showed that SLUG and HIF-1*α* were colocalized in the cytoplasm and nucleus under different conditions (Figure 5(d)), which further confirmed the regulatory effect of HIF-1*α* on SLUG. Our results also showed that both SLUG and HIF-1*α* expression could be downregulated by inositol, which was consistent with the results shown by mRNA-seq and qRT-PCR. Moreover, a qPCR analysis (Figure 5(e)) and western blot analysis (Figure 5(f)) both showed that SLUG expression was significantly upregulated when HIF-1*α* was overexpressed. Taken together, these results indicated that *SLUG* was a direct target of HIF-1*α*. In addition, N-cadherin expression was also dramatically upregulated when HIF-1*α* was overexpressed, while E-cadherin expression was dramatically downregulated when compared to its expression in the vector group (Figure 5(f)). These results indicated that overexpression of *SLUG* induced by HIF-1*α* promoted the EMT process.

### 3.6. Inositol Promoted Autophagy, Which Blocked EMT by Regulating SLUG Expression

**Figure 6:**
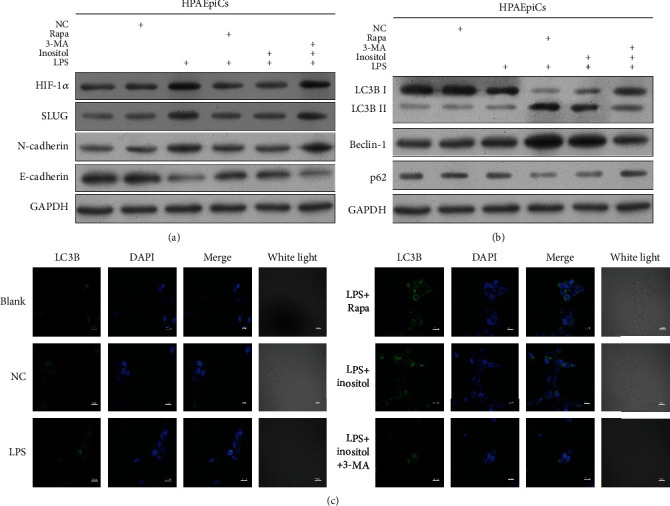
Inositol promoted autophagy, which blocked EMT by regulating SLUG expression. **(**a) SLUG, HIF-1*α*, N-cadherin, and E-cadherin expression in HPAEpiC cells treated with RAPA and 3-MA. (b) Beclin-1, LC3BI, LC3BII, and P62 expression in cells treated with RAPA and 3-MA; (c) LC3B expression was detected by immunofluorescence and DAPI was used to stain the nucleus, Bar = 20 *μ*m.

In order to further investigate the effect of inositol on the autophagy that contributes to EMT by SLUG expression, a rescue experiment was performed in which cells were treated with RAPA and 3-MA. As shown in Figure 6(a), the levels of SLUG and HIF-1*α* expression were significantly elevated in the LPS group when compared with both the blank control group and NC group. However, those increases were attenuated when the LPS-induced cells were treated with RAPA or inositol. A further analysis showed that the levels of SLUG and HIF-1*α* expression in LPS-induced cells were partially recovered when the cells were treated with 3-MA. Furthermore, the changes in N-cadherin expression that occurred in the different groups showed the same trends as the changes in SLUG and HIF-1*α* expression, while the changes in E-cadherin expression showed the opposite trends, suggesting that inhibition of autophagy by 3-MA could partially reverse the deactivation of EMT caused by inositol (Figure 6(a)). Changes that occurred in the expression of autophagy-related genes in the different groups were also detected. The levels of Beclin-1 and LC3BII expression were significantly increased in the RAPA and inositol group when compared to the NC group, while the levels of Beclin-1 and LC3BII expression in the LPS with inositol group were decreased after treatment with 3-MA. However, the opposite changes in LC3BI and P62 expression occurred in the different groups (Figure 6(b)). In addition, IF analysis showed that the immunofluorescence signal of LC3B was significantly stronger in LPS-induced cells treated with RAPA or inositol. However, the immunofluorescence signal of LC3B in the LPS with inositol group was dramatically decreased by treatment with 3-MA (Figure 6(c) and Supplementary Figure 1C). These results demonstrated that inositol promoted autophagy which blocked EMT by regulating SLUG expression.

## 4. Discussion

ARDS is a heterogeneous syndrome with complex pathology and pathogenesis [19], and there is still no clear and effective treatment strategy. Therefore, it is of great importance to find new substances for treating ARDS. More importantly, the effective remission of acute respiratory distress syndrome- (ARDS-) caused pulmonary fibrosis determines the recovery of lung function. Fortunately, our results showed that inositol alleviates pulmonary fibrosis by promoting autophagy via inhibiting the EMT process and SLUG expression in ARDS. This finding provides an important foundation for using inositol in treatment of ARDS.

Inositol is a small polyol compound that is widely distributed in organisms. It is not only a basic substance of life but also serves as an important intracellular signaling molecule that participates in all important aspects of life activities, including regulation of gene expression, phosphorus storage, growth hormone and receptor connection, membrane binding, stress resistance, and apoptosis regulation [20]. Increasing numbers of studies have also indicated that inositol plays crucial roles in acute lung injuries and ARDS. In this study, we found that the degrees of inflammatory cell infiltration, pulmonary hemorrhage, and collagen deposition that occurred in ARDS model mice could be reduced by treatment with inositol. Moreover, a further analysis showed that the elevated levels of IL-1*β*, IL-6, IL-17, and MCP-1 in the ARDS model mice could be decreased by treatment with inositol. These findings indicated that inositol can attenuate lung injuries by reducing an increase in inflammatory factors.

Autophagy is an evolutionarily highly conserved starvation response mechanism that plays an important role in biological development and the maintenance of body homeostasis. Autophagy participates in the pathological process of various diseases, such as neurodegenerative diseases, cardiovascular and cerebrovascular diseases, tumors, and lung injuries. Regulating cell autophagy has become an important strategy for preventing and treating these diseases. Increasing evidence indicates that inositol participates in regulating autophagy. For example, inositol-6 phosphate was shown to induce autophagy by inhibiting the Akt/mTOR pathway, which led to the autophagy-mediated death of HT-29 colon cancer cells [21]. Abnormal inositol levels caused by impaired *GCR1* transcription led to vacuole formation and autophagy in *Saccharomyces cerevisiae* [22]. Deng et al. demonstrated that inositol pyrophosphates mediated the hypoxia-induced autophagy and apoptosis of BM-MSCs by attenuating activation of the Akt/mTOR signaling pathway [23]. In our current study, LC3B immunofluorescence staining and changes in the numbers of autophagosomes demonstrated that inositol could aggravate autophagy in both ARDS cells and the ARDS model mice. Furthermore, inositol and an autophagy activator had basically the same effect on autophagy in ARDS model cells, and an autophagy inhibitor (3-MA) was found to partially reverse the effects of inositol on autophagy in an ARDS cell model. These results demonstrated that inositol can attenuate lung injuries by activating autophagy.

Lung cell EMT plays an important role in ventilation-associated lung fibrosis, which contributes to the poor outcomes of patients with ARDS. Numerous studies have indicated that inhibition of the EMT process can ameliorate pulmonary fibrosis [24]. Those studies have also shown that inositol suppresses cell motility and invasiveness by inducing cytoskeletal modifications that induce an EMT reversion in breast cancer cells [25], suggesting that inositol can inhibit the EMT process. However, there has been no report concerning the effect of inositol on the EMT that occurs in ARDS. In this study, we demonstrated that N-cadherin expression was significantly elevated in the ARDS model groups when compared with the blank control groups. However, the increase in N-cadherin expression induced by LPS could be attenuated by treatment with inositol. Furthermore, the changes in E-cadherin expression showed the opposite trend when compared with the changes in N-cadherin expression that occurred in different groups, demonstrating that inositol could inhibit the EMT process in ARDS [25]. SLUG (Snail-2) and HIF-1*α* are key factors that play crucial roles in the EMT process [26]. Our mRNA sequencing and qRT-PCR analyses showed that SLUG was significantly upregulated in the ARDS model group but could be downregulated by treatment with inositol, suggesting that inositol can inhibit the EMT process in the ARDS models by downregulating SLUG expression. In fact, it has been shown that HIF-1*α* can regulate paraquat poisoning-induced early pulmonary fibrosis by regulating EMT via the Snail and *β*-catenin pathways [27]. Consistent with that finding, we investigated whether HIF-1*α* could directly bind to *SLUG* at loci 776-783 in its promoter region. In addition, we demonstrated that N-cadherin expression was dramatically upregulated when HIF-1*α* was overexpressed, while E-cadherin expression was dramatically downregulated when compared with its expression in a vector blank control group. These results indicated that inositol blocked the EMT process by inhibiting HIF-1*α* -mediated SLUG expression.

EMT-related signal pathways have an impact on autophagy; conversely, autophagy activation can suppress or strengthen EMT by regulating various signaling pathways. Inhibition of EMT by use of autophagy inhibitors or activators might be a novel strategy for treating cancers [28]. It was recently shown that autophagy inhibition-mediated EMT could augment local myofibroblast differentiation via p65/RELA-mediated transactivation of Snail2 caused by increased p62/SQSTM1 expression in pulmonary fibrosis, suggesting that autophagy could inhibit EMT to extenuate pulmonary fibrosis [29]. Our current study showed that N-cadherin expression in RAPA or inositol-treated ARDS cells could be elevated by treatment with 3-MA, which was similar to the trends for SLUG and HIF-1*α* expression, while the opposite changes occurred for E-cadherin expression. This suggested that inhibition of autophagy by 3-MA could partially reverse the inhibition of EMT caused by inositol.

## 5. Conclusion

This study explored the role and mechanism of myo-inositol in the development of ARDS. We used an LPS-induced ARDS cell model and mouse model to prove that inositol could alleviate pulmonary fibrosis caused by ARDS. Studies conducted with an autophagy activator and autophagy inhibitor showed that inositol could activate autophagy in an ARDS cell model. Meanwhile, we found that the transcription factor HIF-1*α* could regulate SLUG expression, and that SLUG could regulate the key gene E-Cadherin involved in the EMT process and N-cadherin expression, which are both regulated by inositol. Therefore, our results suggest that inositol alleviates pulmonary fibrosis by promoting autophagy via inhibiting the EMT process mediated by the HIF-1*α*-SLUG axis in ARDS.

## Data Availability

All data generated or analyzed during this study are included in this published article.

## Supplementary Materials

Supplementary Materials Supplementary Figure 1: fluorescence intensity analysis. Supplementary Figure 2: mapping of the genome for the sequencing reads from different samples. Supplementary Figure 3: scatter plot of GO and KEGG enrichment of differentially expressed genes.
