# Supplementary material for: Inositol Alleviates Pulmonary Fibrosis by Promoting Autophagy via Inhibiting the HIF-1α-SLUG Axis in Acute Respiratory Distress Syndrome
Source: Oxid Med Cell Longev. 2022 Dec 23;2022:1030238. doi: 10.1155/2022/1030238 (PMC9803570; doi:10.1155/2022/1030238)
Supplement: Supplementary Materials — Supplementary Figure 1: fluorescence intensity analysis. Supplementary Figure 2: mapping of the genome for the sequencing reads from different samples. Supplementary Figure 3: scatter plot of GO and KEGG enrichment of differentially expressed genes. [file 1030238.f1.docx]

**Supplementary Figure 1.**


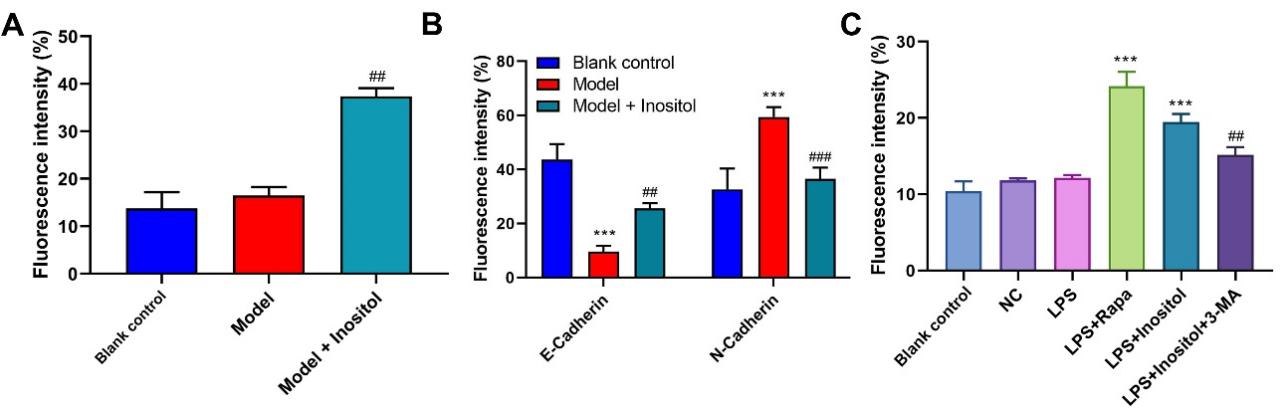


**Fluorescence intensity analysis.** A, Fluorescence intensity analysis of LC3B was performed to detect autophagy changes in LPS-induced HPAEpiCs with and without inositol treatment. Data represents the mean value ± SD, #, Model with Inositol group *vs.* Model group: ## p<0.01. B, E-cadherin and N-cadherin fluorescence intensity were detected. Data represents the mean value ± SD, *, Model group vs. Blank control group: ***, P<0.001; #, Model with Inositol group *vs.* Model group: ##p<0.01; ###p<0.001. C, Fluorescence intensity analysis of LC3B was performed. Data represents the mean value ± SD, *, LPS + Rapa or LPS + Inositol vs. LPS group; ***, *P* < 0.001; #, LPS + Inositol + 3-MA group vs. LPS + Rapa or LPS + Inositol group: ##p<0.01

**Supplementary Figure 2.**


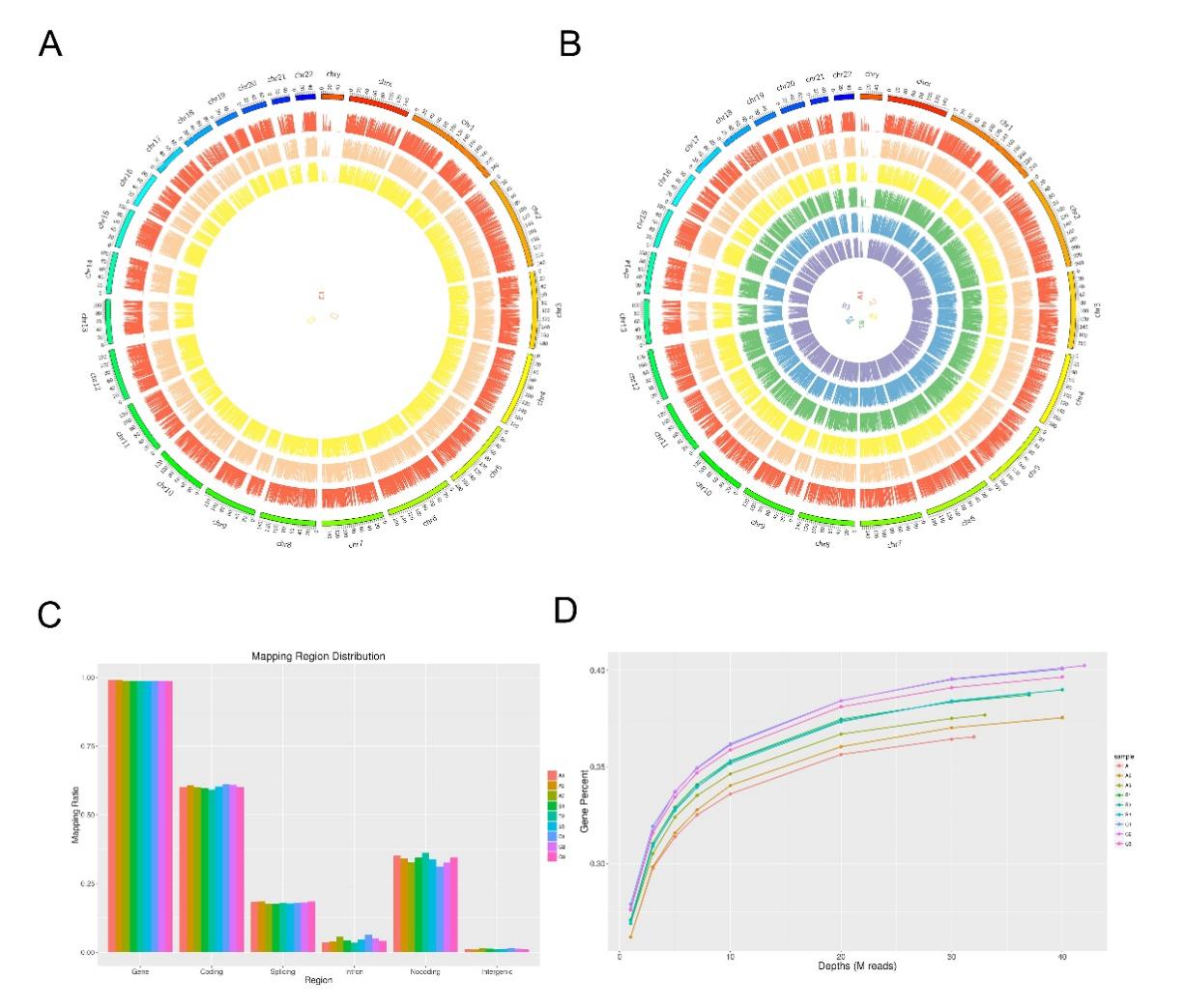


**Mapping of the genome for the sequencing reads from different samples.** A and B, Genomic coverage map. A 1K window was used to obtain a coverage distribution for the genome. The outermost circle in the figure is the genome, and each inner circle represents the chromosome coverage of a sample. C, Region’s distribution of the reads. Figure shows the ratio of the measured reads aligned with the gene region, coding region, splice site, intron, and non-coding region. D, Saturation analysis graph. The abscissa is the amount of data sequenced and the ordinate is the number of genes covered, which indicates whether the number of sequences is sufficient.

**Supplementary Figure 3**

**
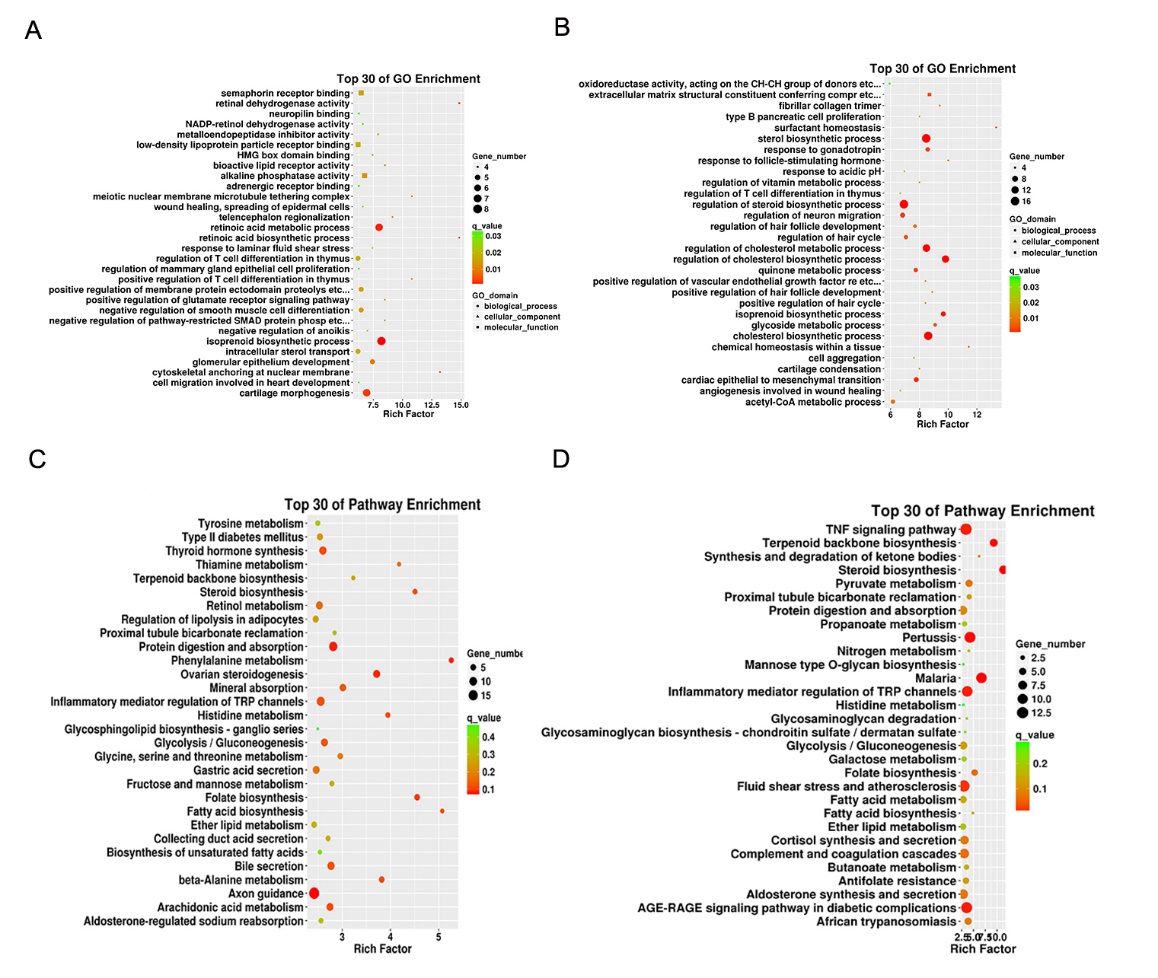
**

**Supplementary Figure 3.** **Scatter plot of GO and KEGG enrichment of differentially expressed genes.**

A, GO enrichment scatter plot of genes that were differentially expressed in the model group vs. the Blank control group. B, GO enrichment scatter plot of genes that were differentially expressed in the model with inositol group vs. the model group. The greater the rich factor, the greater the degree of enrichment. Q-value is the p-value after multiple hypothesis testing correction has been completed; the smaller the value, the more significant the enrichment. C, KEGG (pathway) enrichment scatter plot of genes that were differentially expressed in the model group vs. the Blank control group. D, KEGG (pathway) enrichment scatter plot of genes that were differentially expressed in the model with inositol group vs. the model group. The greater the rich factor, the greater the degree of enrichment. Q-value is the p-value after multiple hypothesis testing correction has been completed; the smaller the value, the more significant the enrichment.
